# Binary Toxin Subunits of *Lysinibacillus sphaericus* Are Monomeric and Form Heterodimers after *In Vitro* Activation

**DOI:** 10.1371/journal.pone.0158356

**Published:** 2016-06-24

**Authors:** Wahyu Surya, Sivadatch Chooduang, Yeu Khai Choong, Jaume Torres, Panadda Boonserm

**Affiliations:** 1 School of Biological Sciences, Nanyang Technological University, Singapore, Singapore; 2 Institute of Molecular Biosciences, Mahidol University, Salaya, Phuttamonthon, Nakhon Pathom, Thailand; 3 Persiaran Universiti, Putra Nilai, Nilai, Negeri Sembilan, Malaysia; University of Tennessee, UNITED STATES

## Abstract

The binary toxin from *Lysinibacillus sphaericus* has been successfully used for controlling mosquito-transmitted diseases. An activation step shortens both subunits BinA and BinB before their interaction with membranes and internalization in midgut cells, but the precise role of this activation step is unknown. Herein, we show conclusively using three orthogonal biophysical techniques that protoxin subunits form only monomers in aqueous solution. However, *in vitro* activated toxins readily form heterodimers. This oligomeric state did not change after incubation of these heterodimers with detergent. These results are consistent with the evidence that maximal toxicity in mosquito larvae is achieved when the two subunits, BinA and BinB, are in a 1:1 molar ratio, and directly link proteolytic activation to heterodimerization. Formation of a heterodimer must thus be necessary for subsequent steps, e.g., interaction with membranes, or with a suitable receptor in susceptible mosquito species. Lastly, despite existing similarities between BinB C-terminal domain with domains 3 and 4 of pore-forming aerolysin, no aerolysin-like SDS-resistant heptameric oligomers were observed when the activated Bin subunits were incubated in the presence of detergents or lipidic membranes.

## Introduction

The control of mosquitoes with pesticides is necessary to prevent mosquito-borne diseases such as dengue, malaria, filariasis or Japanese encephalitis [1, 2]. However, synthetic pesticides cause negative health effects on humans and other organisms, whereas mosquitoes are prone to develop resistance to these chemicals [3–5]. In contrast, development of bioinsecticides, e.g., derived from bacteria, have been successful in mosquito control programs for a long time. Bioinsecticides do not disturb the environment, and exhibit high specificity to insect targets [6, 7]. *Lysinibacillus sphaericus (sp*.*)* [8] (previously known as *Bacillus sphaericus*) is a Gram-positive spore-forming bacterium that is able to produce insecticidal toxins against mosquito larvae [8, 9]. *L*. *sphaericus* produces two proteins, BinA (42 kDa) and BinB (51 kDa), which together form the binary toxin (Bin) [10, 11]. The pro-toxins, hereafter referred to as pro-A and pro-B, appear during the sporulation phase, and accumulate as crystalline inclusions [12]. The binary toxin is highly active against *Culex sp*., moderately toxic to *Anopheles sp*. and less toxic or non-toxic to *Aedes sp*. [13]. It is currently accepted that after ingestion by larvae, pro-A and pro-B are processed by larval gut proteases and become ‘activated’ to produce 39 kDa (act-A) and 43 kDa (act-B) subunits [10, 11]. The activated BinB (act-B) has been shown to specifically bind to a GPI-anchored maltase 1 receptor (Cpm1 in *Culex* species) on brush border membranes of susceptible larval midgut [14–17]. This may help BinA to target the membrane, and to provide toxicity to the complex [18]. Upon binding, the binary toxin is thought to internalize into the target cells by an unknown mechanism, eventually causing larval death [19].

Bin toxins were first purified in 1985 by Baumann et al. [20] who, based on major charge differences between the two subunits, suggested that formation of an A-B heterodimer was likely. This was supported by the fact that maximum activity against mosquito larvae was obtained when mixing the two components in a 1:1 molar ratio [21]. However, the precise nature of the oligomers, if any, formed by the subunits before and after activation is still controversial. Earlier studies show striking discrepancies, e.g., formation of heterotetramers (BinA_2_-BinB_2_) for protoxins and no interaction of their activated subunits [22], complete formation of 1:1 complexes between activated subunits at low μM concentration [23], or weak interaction between activated subunits, with k_a_ ~5 × 10^3^ M^−1^ measured by surface plasmon resonance (SPR), i.e., a 5 mM activated toxin would produce 50% heterodimer [24].

Insights into Bin toxins oligomerization behavior may be found in the recent three-dimensional structure of act-B solved by X-ray crystallography [25]. In the latter, a globular N-terminal domain reminiscent of lectins has been proposed to be involved in receptor recognition. The C-terminal domain shares similar three-dimensional folding with aerolysin type β-pore forming toxins, despite a low sequence identity, with a cluster of aromatic residues exposed on its surface, suggested to constitute a potential transmembrane domain, and a predominance of Ser/Thr on the outer face of its β-sheets, similar to domains 3 and 4 in aerolysin and other aerolysin type β pore-forming toxins (β-PFTs) [26]. Incidentally, BinB has been shown to insert into model lipid bilayers [27, 28], and its C-terminal domain has been proposed to be pore-forming [29]. Aerolysin, both before and after activation, forms homodimers in a reversible equilibrium with monomers in aqueous solution [30, 31], whereas a hydrophobic environment suffices to trigger SDS-resistant heptameric oligomers [32, 33]. Hence, we hypothesize, based on the observed structural similarities between BinB (C-terminal domain) and domains of aerolysin, that the cytolytic mechanism for Bin toxins may be similar to that of aerolysin. In consequence, large aerolysin-like SDS-resistant BinA or BinB oligomers may be observed. Thus, the objective of the present paper is to precisely determine the oligomeric behavior of Bin toxins before and after activation, and the behavior of the activated toxin in presence of hydrophobic environments.

## Materials and Methods

### Expression of protein

His-tagged proteins pro-A and pro-B were expressed in soluble form in *E*.*coli* BL21(DH3) pLysS after induction with 0.2 mM IPTG at 18°C for 5 h [23]. Harvested cell pellets were resuspended in lysis buffer (50mM Tris-HCl, 200 mM NaCl, pH 8.0) and lysed with a sonicator. The cell lysate was centrifuged at 15,000 g for 1 h and the supernatant was collected and filtered through a 0.2 μm syringe filter before purification.

### Protein purification and overall secondary structure analysis

A two-step purification was performed using affinity and size exclusion chromatography. A HiTrap^™^ Chelating HP (GE healthcare, Uppsala, Sweden) column was precharged with 0.1 M NiSO_4_ and equilibrated with lysis buffer. The supernatant collected from the cell lysate (see above) was loaded into the column. Washing was performed with lysis buffer containing 25 mM imidazole and later with 50 mM imidazole, as this increased the purity of the final product. Bound (His)_6_-tagged protein was eluted with lysis buffer containing 100 mM imidazole. Protein-containing fractions were pooled and concentrated by ultrafiltration at 4°C using a Centriprep column (30-kDa cutoff for pro-A or -B). Protoxin samples were stored at -80°C. Toxicity was tested against larvae immediately before the experiments or before activation, and showed the similar LC_50_ as the fresh toxin (2–6 ng/mL). To obtain activated toxin, trypsin (EC 3.4.21.4) was added to a trypsin-to-toxin mass ratio of 1:10 and incubated at 37°C for 2 h. The reaction was terminated by phenylmethylsulfonyl fluoride (PMSF) addition and the cleaved –activated—protein was purified by size-exclusion chromatography (Superdex 200 HR 10/30 column, GE Healthcare Life Science, Uppsala, Sweden). This column was equilibrated with 50 mM Tris-HCl pH 9.0, 50 mM NaCl, and 1 mM DTT. The toxins, either protoxins (pro-A and pro-B) or activated (act-A and act-B) were mixed at 1:1 molar ratio, incubated at room temperature for 15 minutes, and injected into the Superdex 200 column. In all cases, the flow rate was 0.4 mL/min. Protein quality and quantity was estimated by SDS-PAGE and a Bradford assay, respectively. Overall secondary structure was monitored using circular dichroism (CD, Applied Photophysics, Leatherhead, UK), and CD spectra were analyzed using DichroWeb [34].

### SDS gel electrophoresis

Samples consisting of act-A and act-B (1:1 molar ratio) in 50 mM Tris, 50 mM NaCl, pH 9.0 were prepared in the absence of lipid or detergent, or mixed with 10 mM DPC (n-dodecylphosphocholine, Avanti Polar Lipids, Alabaster, AL, USA), 5 mM C14SB (3-(N,N-dimethylmyristylammonio) propanesulfonate, Sigma, St. Louis, MO), 35 mM C8E5 (pentaethylene glycol monooctyl ether, Sigma, St. Louis, MO), or with liposomes at 1:1, 1:10, and 1:100 protein-to-lipid ratio. Liposomes were prepared from a 1:1 (molar ratio) mixture of PA (phosphatidic acid, from egg yolk, Avanti Polar Lipids) and PC (phosphatidylcholine, from egg yolk, Avanti Polar Lipids). SDS sample buffer was added to the sample to a final protein concentration of 1 μg/μL. Where indicated, after vortexing for 1 minute samples were heated at 70°C for 10 minutes prior to loading into NuPAGE Novex 4–12% Bis-Tris gel (Invitrogen, Carlsbad, CA, USA). The loading volume was 5 μL. The gel was subsequently electrophoresed and stained according to the manufacturer's instructions. The sample was electrophoresed at room temperature at a constant voltage of 100 V for 30 min. After completion, the SDS/PAGE gel was first stained with Coomassie blue.

### Static light scattering (SLS) and dynamic light scattering (DLS)

Molecular weight and size of particles in solution was determined by SLS and DLS, respectively, using a Zetasizer Nano-ZS instrument (Malvern, Worcestershire, UK) at 25°C. The Rayleigh ratio was calibrated using toluene, as instructed by the manufacturer. Protein concentrations ranged typically from 0.05 to 1.2 mg/mL. Samples were collected from gel filtration in 50 mM Tris-HCl pH 9.0, 50 mM NaCl, and 1 mM DTT, and filtered through a 0.02 μm Whatman Anotop^®^ 10 syringe filter (GE Healthcare, Germany) before the measurements. The molecular weight was determined from the Debye plot of (KC/R_**θ**_) vs concentration.

### Analytical ultracentrifugation (AUC)

AUC experiments were performed using a Beckman Coulter XL-I protein characterization system at 20°C using an An-50 Ti analytical rotor. For sedimentation velocity (SV) experiments, samples were solubilized in 50 mM Tris-HCl pH 9.0, 50 mM NaCl, and 1 mM DTT, with or without 35 mM C8E5 detergent. The protein concentration was 7 μM in monomers (3.5 μM of each BinA and BinB when mixed), i.e., 280 nm absorbance (A_280_) of 0.5 at 1.2 cm pathlength. Buffer alone was used as reference. Samples were loaded into standard double sector Epon charcoal-filled centerpieces and centrifuged at 45,000 rpm. Typically, 90 scans were collected, one every 10 min. Partial specific volumes of act-A and act-B, 0.72820 and 0.7307 cm^3^/g, respectively, were estimated by using SEDNTERP [35]. A *c(s)* and a *c*(*s*, *ffo*) distribution was obtained with SEDFIT [36].

For sedimentation equilibrium (SE) experiments, six-channel Epon charcoal-filled centerpieces were loaded with three different protein concentrations—0.3, 0.5 and 0.8 absorbance at 280 nm (3–10 μM in monomer concentration), at 1.2 cm pathlength—and run at the following rotor speeds: 10, 12.3, 15, 18.4, and 22.5 krpm. The buffer was the same as that used in SV experiments. Samples exposed to 35 mM C8E5 detergent were density matched with 16% D_2_O, which was determined experimentally [37]. Multi-speed equilibrium data were obtained on absorption mode at 280 nm and were globally fitted in SEDPHAT using the Marquardt-Levenberg algorithm to an A+B heteroassociation model [38].

## Results

### Oligomerization of act-A and act-B assessed by SDS-PAGE after exposure to hydrophobic environments

**Fig 1:**
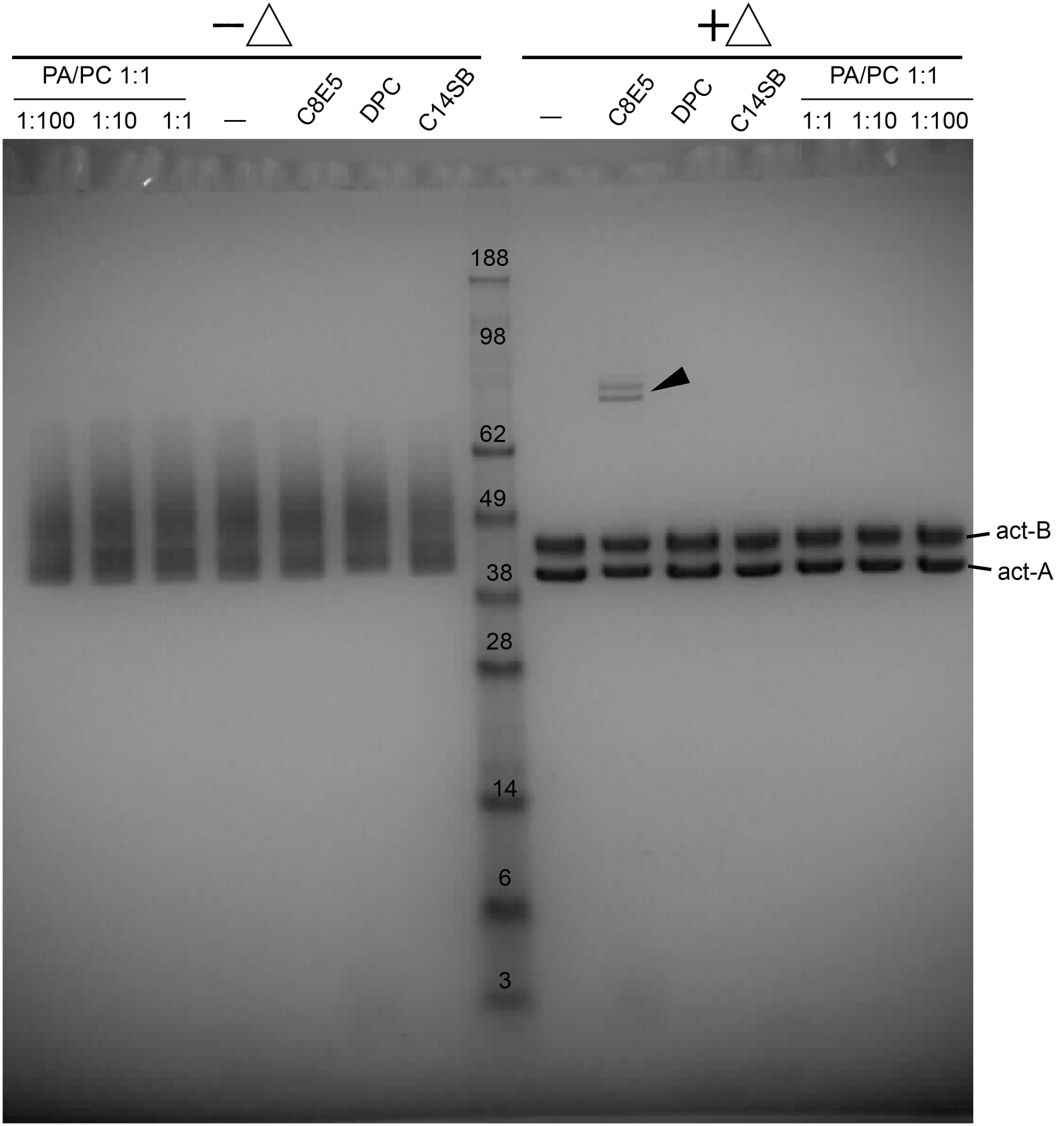
SDS-PAGE of the equimolar mixture act-A/act-B. (A) Equimolar mixture of act-A/act-B incubated in aqueous buffer (─), or in the presence of hydrophobic environments: liposomes (PC:PA) at the protein:lipid ratios shown, or detergents indicated. After SDS solubilization the samples were either heated (+Δ) or not heated (-Δ). The migration of monomeric act-A and act-B is indicated. All the lanes contain identical protein quantity (5 μg). The black arrows point to faint bands that correspond to a small proportion of homodimers or heterodimers.

The observed structural similarities between domains 3 and 4 of aerolysin and the C-terminal domain of BinB [25] suggest a somewhat similar mechanism of action for these toxins. Although both pro-aerolysin and aerolysin migrate as monomers in SDS, previous exposure to a hydrophobic environment, i.e., liposomes or non-ionic detergent polyethylene glycol monooctyl ether (octyl-POE), produced exclusively SDS-resistant heptameric oligomers [33]. To test if a similar behavior is observed in Bin toxins, an equimolar mixture of act-A and act-B was exposed to either PA/PC liposomes or detergents C14SB, DPC, and C8E5, and subsequently solubilized in SDS for electrophoresis. However, act-A and act-B only formed monomers in SDS (Fig 1). The results with and without heating were similar, although the bands appeared more compact in the former condition. The sample heated in C8E5 showed very faint bands (see arrow) that may correspond to a small percentage of homo and/or heterodimers. Therefore, in contrast with aerolysin, neither act-A or act-B, separately or as an equimolar mixture, form any SDS-resistant oligomers when exposed to hydrophobic environments, including lipid bilayers.

### Oligomerization of binary toxin subunits in aqueous solution—gel filtration

**Fig 2:**
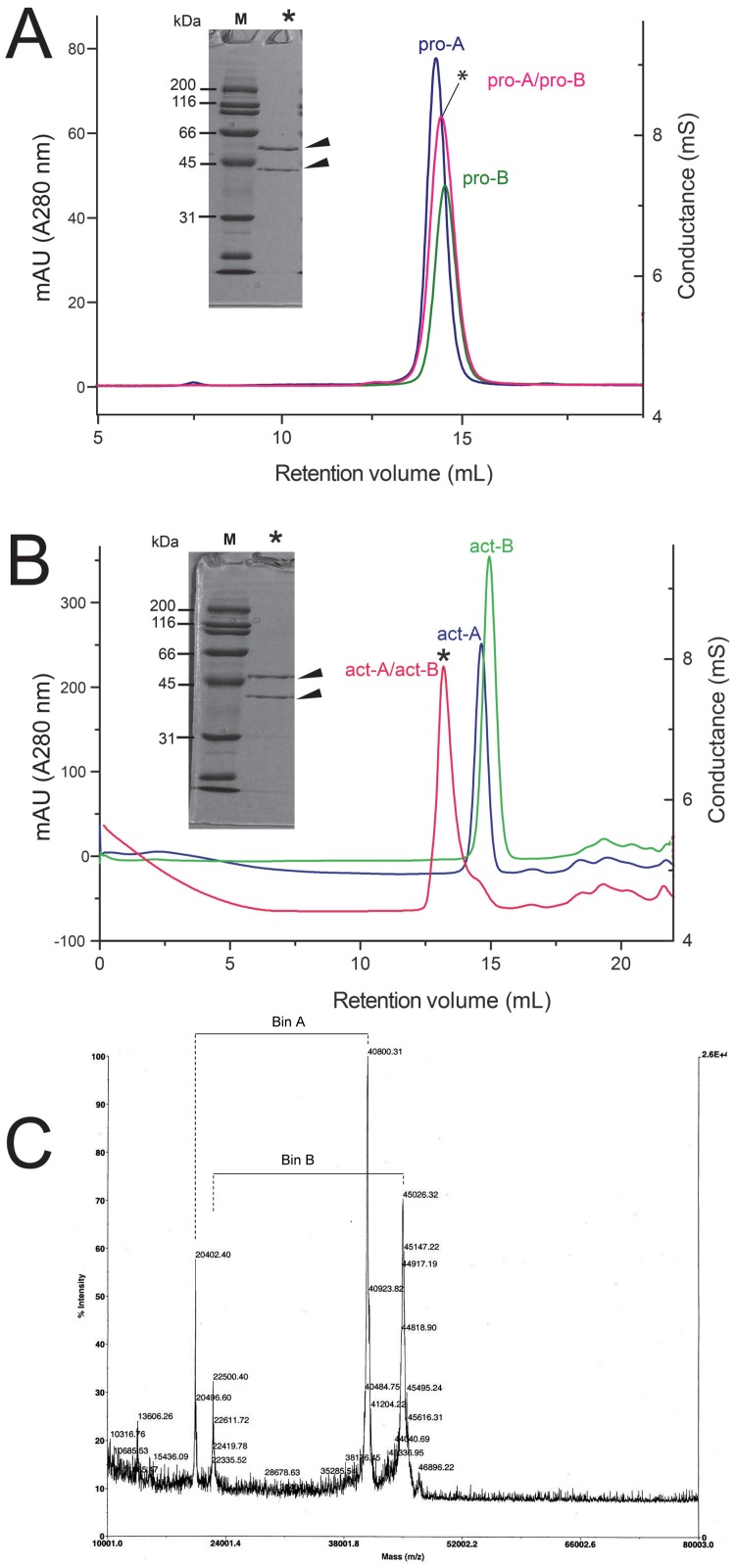
Gel filtration of pro- and act-Bin subunits. (A) Elution profile of pro-A, pro-B, and their mixture (1:1 molar ratio, *); (B) the same as (A) for act-A and act-B. Protein concentration before and after chromatography was typically 60 μM and 18 μM (total monomer), respectively. The SDS PAGE gel for the elution band of the 1:1 mixture is shown in the insert; (C) MALDI-TOF mass spectrum of the elution band shown in B. Four peaks are observed, associated with masses of approximately 40 kDa (mass to charge ratios (m/z) of 40,800 and 20,402 Da) and 45 kDa (m/z of 22,500 and 45,026 Da).

To study the oligomerization behavior of Bin toxin subunits without detergent, their elution profile was monitored using gel filtration. The pro-toxins, pro-A and pro-B, and their 1:1 mixture, eluted with almost overlapped elution volumes, between 14.5 and 14.8 mL (this volume corresponds to globular proteins of ~ 60 kDa) (Fig 2A). The fact that the bands overlap, and that the single elution band observed for the 1:1 mixture contained both pro-toxin subunits in a 1:1 molar ratio (Fig 2A, insert), suggests that there is no oligomerization in any of these samples.

The individual activated subunits (Fig 2B), act-A and act-B, eluted at 14.8 and 15 mL respectively. This is the elution volume of ~ 50 kDa globular proteins, therefore it is consistent with monomers. The mixture act-A/act-B, in contrast, eluted as a single elution band at 13.2 mL, which corresponds to ~90 kDa for globular proteins. The eluted fraction in the 1:1 mixture contained both subunits (act-A and act-B) in a 1:1 molar ratio (Fig 2B, insert and Fig 2C). Overall, these results show that the two Bin toxin subunits tend to form heterodimers, but only when proteolytically activated.

### Oligomerization of binary toxin subunits in aqueous solution—static and dynamic light scattering (SLS and DLS)

**Fig 3:**
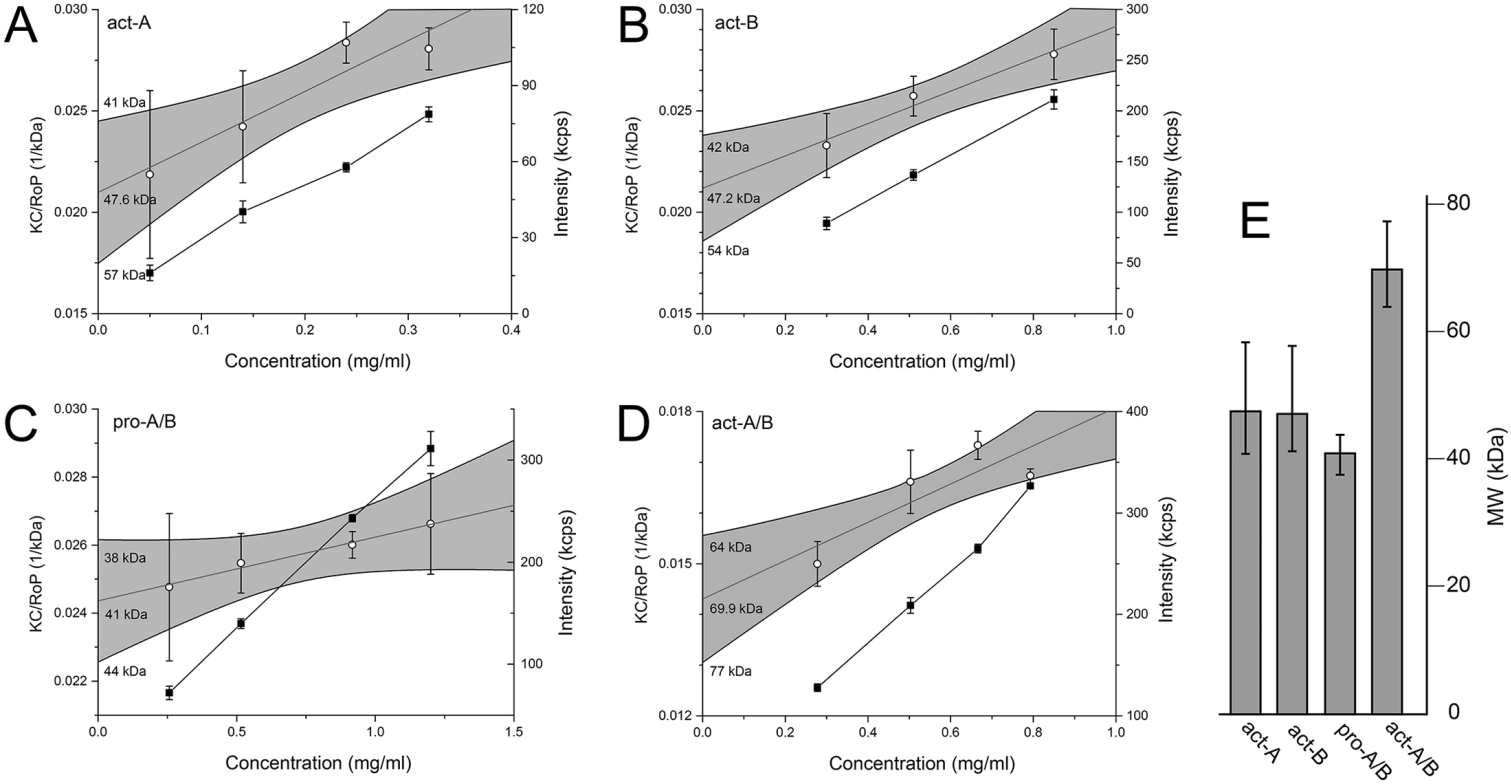
SLS molecular weight determination of BinA and BinB in aqueous solution. Debye plots of scattered intensity (**■**) and KC/RoP (○) plotted against protein concentration. (A) act-A; (B) act-B; (C) 1:1 mixture of pro-A and pro-B; (D) 1:1 mixture of act-A and act-B; (E) Calculated weight-average molecular weight, where bars represent 95% confidence interval as shown in panels A-D (greyed area, with limit values indicated.). The molecular weight was obtained from the inverse of the Y-intercept of the linear fit of KC/RoP. Experiments were done in triplicate (each data point represents the mean ± SD).

Light scattering was used to determine the molecular weight and size of the species in these samples. The activated toxins act-A and act-B, independently, produced similar molecular weight values (~47 kDa), consistent with monomers (Fig 3A and 3B). The equimolar mixture of the protoxins, pro-A and pro-B produced a weight-average molecular weight of 41 ± 1.3 kDa (Fig 3C). This is close to the average ~45 kDa expected for protoxin monomers and far from the 93 kDa expected for a heterodimer, therefore this is consistent with the gel filtration results shown in Fig 2A. In contrast, mixing the two activated subunits, act-A and act-B, in a 1:1 molar ratio produced a molecular weight of ~70 kDa (Fig 3D), consistent with heterodimer formation. This confirms the conclusions from the gel filtration experiments shown in Fig 2B, and the SLS results are summarized in Fig 3E.

**Fig 4:**
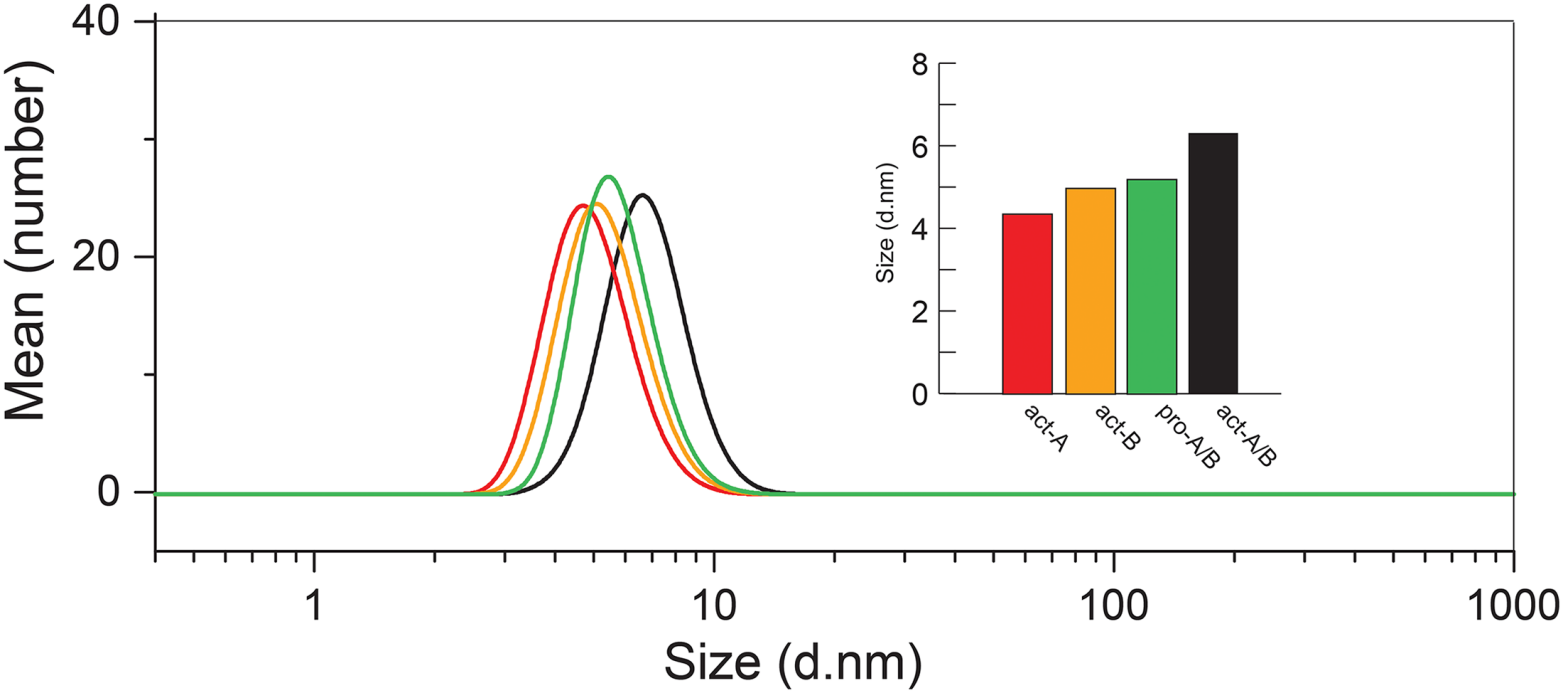
DLS particle size determination of BinA and BinB in aqueous solution. Average size distribution (by particle number) of act-A, act-B, and 1:1 mixtures of pro-A and pro-B, or act-A and act-B, as indicated. The distributions are averaged from at least 5 separate experiments. The insert shows the average size obtained, with the same color code as the size distribution curves.

To test if the weight-average molecular weight (Fig 3) represents a heterogeneous population or a narrow distribution, DLS was used to determine particle size distribution. Each of the samples in Fig 3 produced a single narrow band in the particle number representation (Fig 4), indicative of monodisperse samples. Therefore, the determined molecular weight (Fig 3E) represents that of a narrow size distribution, and not an average of a size-heterogeneous population. The diameters of act-A and act-B were 4.3 and 5 nm, respectively, whereas in their 1:1 mixture, the diameter increased to 6 nm (Fig 4, insert). For the pro-toxin mixture the size was in between these values, 5.3 nm, consistent with their lack of oligomerization.

### Analytical ultracentrifugation (AUC)

**Fig 5:**
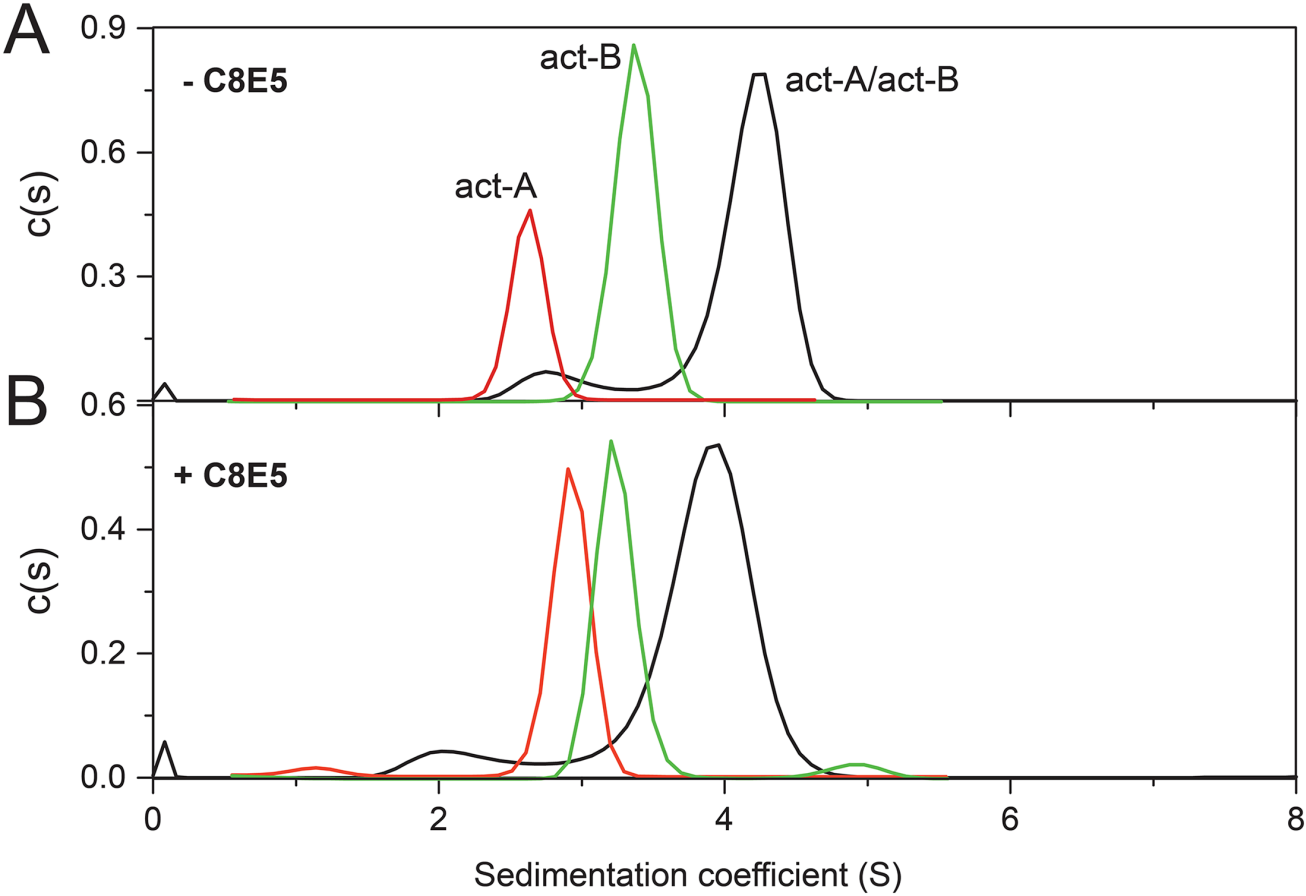
Sedimentation velocity size distribution analysis of act-A and act-B. (A) Sedimentation velocity c(s) distribution of act-A, act-B, and act-A/B mixture (1:1 molar ratio) without detergent; (B) same as (A) in presence of 35 mM detergent C8E5. Samples were centrifuged at 45,000 rpm and the absorbance was measured at 280 nm.

The oligomerization behavior of the activated Bin subunits in absence and presence of detergent was compared using sedimentation velocity (SV) and sedimentation equilibrium (SE). In the absence of detergents (Fig 5A) c(s) plots were centered at ~2.7 S and 3.4 S for act-A and act-B, respectively. The result for act-B is comparable with the theoretical estimate, 3.2 S, obtained using the Hydropro prediction tool [39] from the available crystal structure of the act-B monomer [25]. This value obtained by SV is therefore consistent with act-B being monomeric. Since the value for act-A is even smaller, both activated subunits appear to form monomers in these conditions. However, in the equimolar mixture the c(s) distribution shifted significantly to 4.3 S, consistent with an increase in either overall mass or compactness. Since the latter would require that both subunits change their conformation independently, the most likely explanation is that the increase in S is due to complete formation of heterodimers. Non equimolar models of interaction, e.g., 1:2 or 1:3, would leave some act-B or act-B in monomeric form, but this was not observed in the act-A/act-B mixture. Therefore, these SV results are consistent with the gel filtration and light scattering data (Figs 2–4).

To test if contact with, or insertion in, hydrophobic environments triggers some conformational change leading to formation of larger oligomers, similar SV experiments were performed in the presence of detergent C8E5 (Fig 5B). This detergent is essentially identical to octyl-POE, which was sufficient to trigger aerolysin heptamerization [32, 33]. Results were almost identical to those without detergent, although the S value of act-B (3.2S) and the 1:1 mixture (4S) were slightly lower. Because of the higher density and size of the resulting particle, the presence of detergent should in principle increase the S value. Therefore, the lower S observed in detergent for act-B and mixture act-A/B suggests a conformational change to form a less compact structure. This was not observed for act-A, which increased its S value. These results are consistent with act-B changing its conformation to a more elongated shape upon interaction with membranes, whether or not it interacts with act-A. In turn, the fact that we observed changes in S value for all three species indicates that all of them interacted with the micelles in one way or another. Indeed, even the protoxins suffer a dramatic conformational change when mixed with membranes, as observed previously in IR spectra [27].

**Fig 6:**
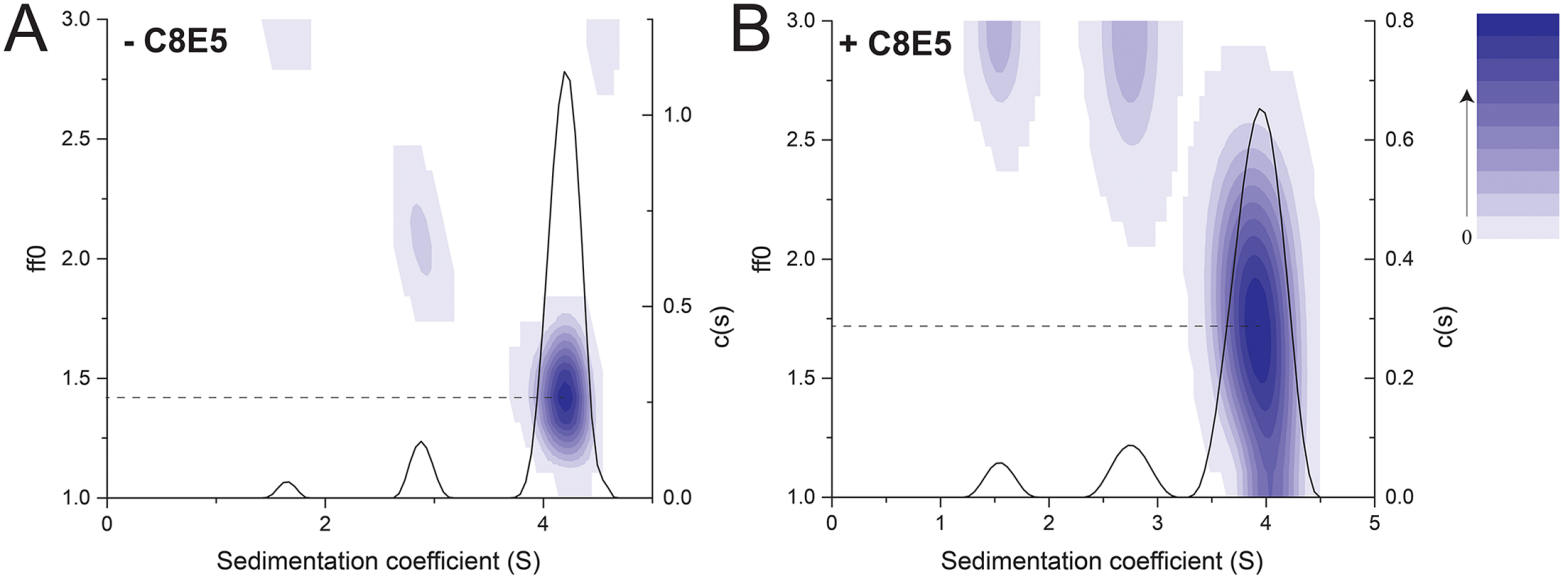
Sedimentation velocity size-and-shape distribution analysis of act-A/B mixture. (A) Sedimentation velocity c(s, f/f0) distribution of actA/actB mixture (1:1 molar ratio) without detergent. The c(s, f/f0) distribution is shown in 2D contours, and the integrated c(s, *) distribution as a solid line; (B) same as (A) in presence of C8E5. The gradient increase step is 0.001510 (A) and 0.000317 (B).

To examine whether a conformational change takes place after interaction with detergent, the SV data was also analyzed with a c(s, f/f0) size-and-shape distribution model (Fig 6). The additional f/f0 dimension represents the frictional coefficient ratio of the sedimenting species relative to that of a spherical particle, and thus reflects the overall shape of the species. The frictional ratios of the heterodimer species in the absence of detergent was 1.4 (Fig 6A) and increased to 1.7 in the presence of detergent (Fig 6B), again confirming that the heterodimer suffers a conformational change to a less globular shape upon contact with, or insertion into, the micellar environment.

**Fig 7:**
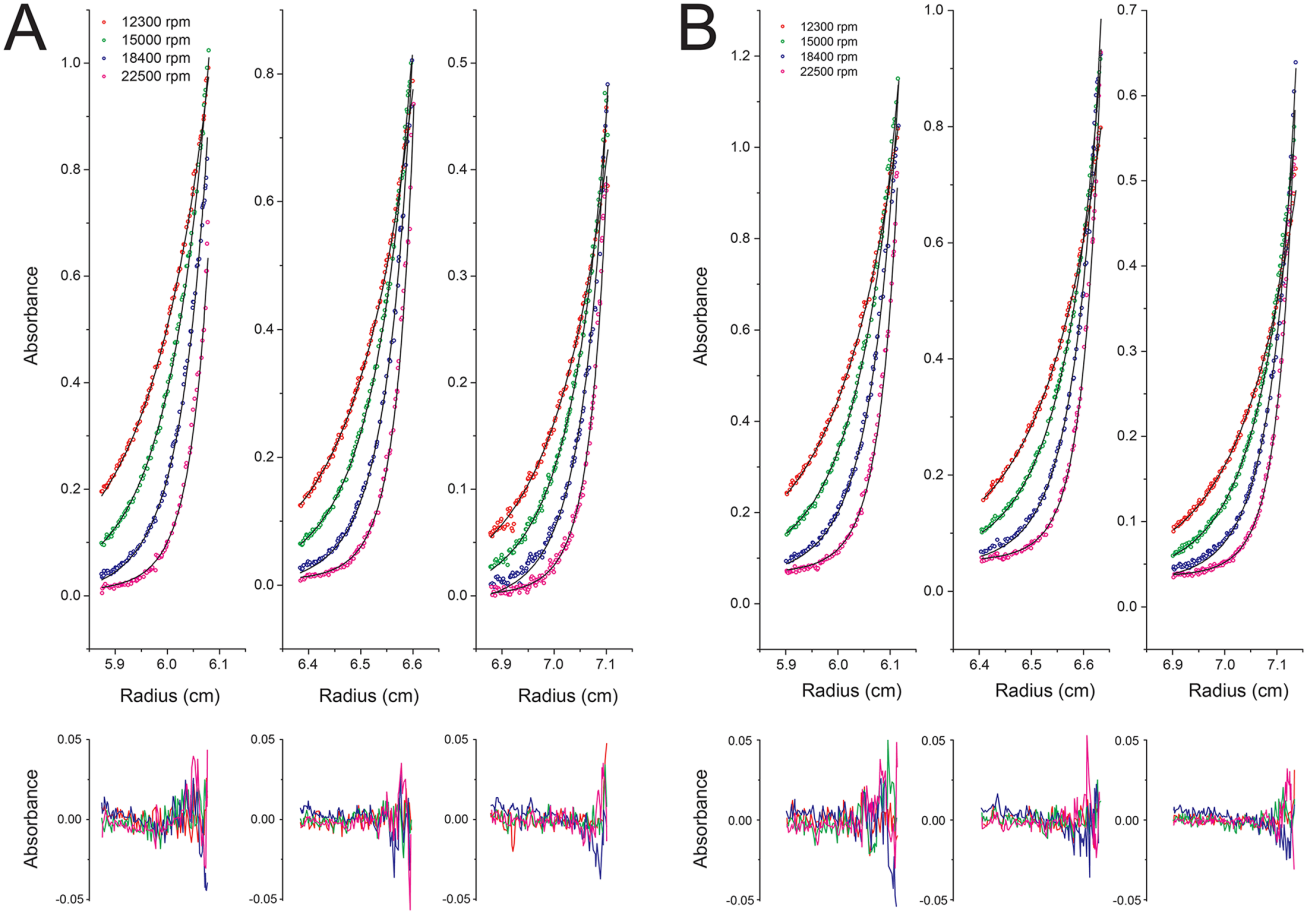
Sedimentation equilibrium of act-B/act-B mixture. Radial distribution profile (open circles) in the absence (A) and presence (B) of detergent C8E5. Data at each speed (12.3, 15,18.4, and 22.5 krpm) are shown in red, green, blue, and purple, respectively. The profiles were fitted to a A+B heteroassociation model (line). Fitting residuals are shown below each plot.

**Fig 8:**
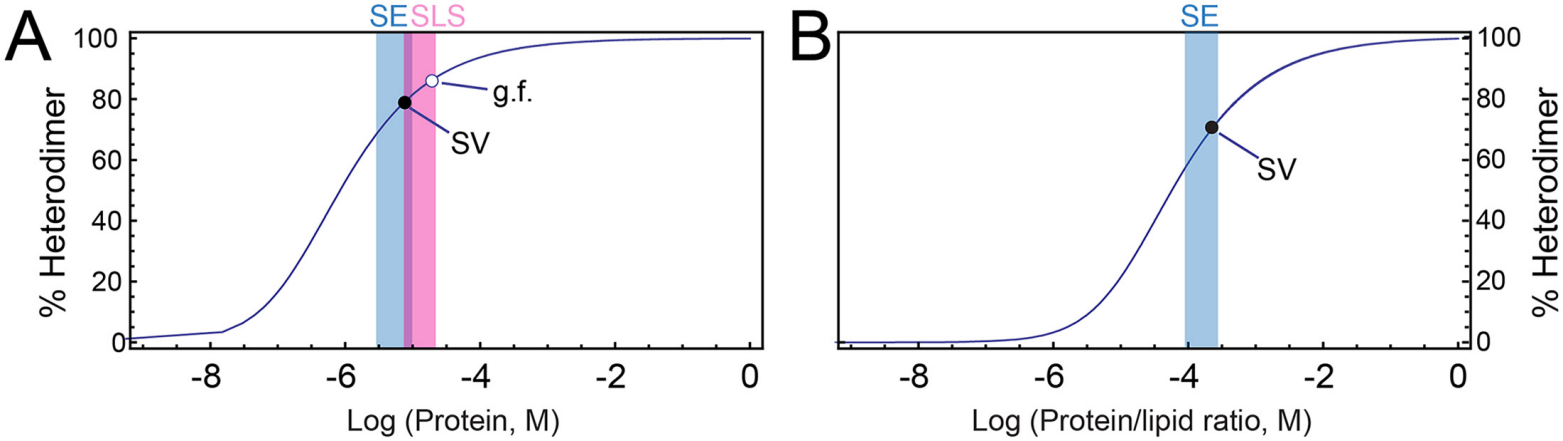
Theoretical heterodimer formation obtained from the association constants (SE). (A) Proportion of heterodimer as a function of protein concentration in the absence of detergent. Blue, range of concentrations used in SE; red, range of concentrations used in SLS (overlap is purple); (○) and (●) are the concentration after gel filtration (1:1 act-A/act-B eluted fraction in Fig 2B) and the concentration used in SV, respectively; (B) proportion of heterodimer in detergent as a function of the protein/detergent molar ratio, calculated as described [40]. Blue has the same meaning as in (A), whereas (○) indicates the lipid/protein ratio used in SV.

Finally, to gain insight into the affinity of the interaction between act-A and act-B, and the molecular weight of the complex formed with and without detergent, we used SE experiments. The data corresponding to the 1:1 mixture act-A/B could be fitted to a reversible heteroassociation model A+B > AB. The affinity constant, K_a_, determined in the absence of detergent was 1.19 × 10^6^ M^-1^ (Fig 7A). This value was used to calculate a curve that represents the proportion of heterodimer as a function of protein concentration (Fig 8A). This figure shows that in the SLS experiments, the % of heterodimer is in the range 80–85%. Thus it is possible that a small proportion of monomer (7–10% of each), contributed to a slight bias to a lower value in the SLS molecular weight calculation. This figure also shows that the SE experiments were performed in the range 68–83% heterodimer. Lastly, at the concentration of the 1:1 act-A/act-B mixture eluted in the gel filtration (Fig 2B), the % of heterodimer should be > 85%, consistent with our results.

In the presence of detergent, the K_a_ was 1.92 × 10^6^ M^-1^. However, this value depends strongly on the detergent concentration, because the volume to consider is the detergent volume in which the protein partitions into, and not the bulk solution. Therefore, assuming that the protein partitions completely into the detergent, with the total C8E5 detergent concentration of 35 mM, and the K_a_ obtained experimentally, the molar fraction association constant (K_x_) [40] was calculated which is independent from the detergent concentration used. Hence, the plot in Fig 8B was obtained from the formula $2K_{x}\cdot x=\frac{y}{{\left(1-y\right)}^{2}}$, where x and y are the protein/detergent molar ratio and the percentage of heterodimer, respectively. This figure shows that the protein/detergent ratio used in SV is at the higher limit of that used in SE, and indicates about ~75% heterodimer, whereas our SV data shows that all the sample is heterodimeric. This deviation is probably due to a non-ideal behavior of our protein [40] but nevertheless, the results are in general consistent with the gel filtration and scattering results.

## Discussion

An earlier study claimed that pro-A and pro-B extracted from bacteria form heterotetramers (BinA_2_-BinB_2_) [22], whereas no interaction was observed between activated subunits, act-A and act-B. These conclusions were largely based on static light scattering data that was not shown, and on bands appeared in SDS electrophoresis, a notably harsh detergent prone to produce artifactual oligomers, especially in membrane interacting proteins. In another report, an equimolar mixture of act-A and act-B eluted in gel filtration as a single band that contained both subunits at 10–20 μM concentration in an equimolar ratio [23], but the precise stoichiometry of the oligomer, e.g., heterodimers (1:1 ratio) or heterotetramers (2:2 ratio) was not determined. Finally, interaction between act-A and act-B in aqueous solution was observed by surface plasmon resonance (SPR) [24]. In this paper, the stoichiometry of this interaction could not be obtained, but association and dissociation rate constants of 4.5 × 10^3^ M^−1^ s^−1^ and 0.8 s^−1^ were measured. These values are equivalent to a K_a_ of 5 × 10^3^ M^−1^, i.e., to observe any interaction, a millimolar concentration of the monomers would be required. We note that these latter experiments used a buffer (50 mM Tris-HCl, pH, 8.0, 100 mM NaCl) different from the one used herein and in a previous report [23], i.e., 50 mM Tris-HCl pH 9.0, 50 mM NaCl. Therefore, a possible reason for the discrepancies is that interaction between act-A and act-B is sensitive to either pH or salt concentration or both.

The heterodimer observed for Bin toxins is reminiscent of the homodimer present in solution for aerolysin. However, our results clearly show that aerolysin and Bin toxins behave very differently. Both pro-aerolysin and activated aerolysin form homodimers in a reversible equilibrium with monomers [30, 31], and crystals of proaerolysin were shown to have unit cells containing homodimers [41, 42]. In contrast, we have shown that, separately, BinA and BinB are monomeric, whether as protoxins or as activated toxins. This is consistent with the monomeric form found in the asymmetric unit of BinB crystal [25]. When the activated subunits are combined however, they readily form a heterodimer. The SV data shows that essentially all the protein forms heterodimers at a concentration of ~7 μM monomer, i.e., 3.5 μM of each activated subunit, and this is not affected by the presence of detergent.

The fact that the maximal toxicity in mosquito larvae is achieved with an equimolar ratio of BinA and BinB [21], and that we have shown that activation leads to heterodimer formation, leads to the hypothesis that this is the form that interacts with its native receptor. This interaction may then trigger other conformational changes or further oligomerization. In any case, the mechanism is not similar to aerolysin because we could not detect SDS-resistant Bin oligomers after mixing the two activated subunits in detergents or in lipid bilayers. The role of the Bin toxin receptor may be to induce a conformational change in the Bin heterodimer upon binding, or just contribute to increase the local toxin concentration to levels that are not accessible experimentally.
